# Large focal pericardial effusion secondary to coronary artery perforation post complex coronary intervention mimics a mass

**DOI:** 10.1093/ehjcr/ytae470

**Published:** 2024-09-02

**Authors:** Christopher N Kanaan, Charles Cannan, Reza Arsanjani, Chadi Ayoub

**Affiliations:** Department of Cardiovascular Medicine, Mayo Clinic, 5777 E Mayo Blvd Phoenix, AZ 85054, USA; Department of Cardiovascular Medicine, Mayo Clinic, 5777 E Mayo Blvd Phoenix, AZ 85054, USA; Department of Cardiovascular Medicine, Mayo Clinic, 5777 E Mayo Blvd Phoenix, AZ 85054, USA; Department of Cardiovascular Medicine, Mayo Clinic, 5777 E Mayo Blvd Phoenix, AZ 85054, USA

## Summary

A new finding of a mass that mimicked a tumour compressing the right atrium was found on multimodality imaging evaluation to be subacute haematoma. This case highlights the potential for delayed presentation of coronary artery perforation (CAP) after complex percutaneous coronary intervention (PCI) and successful conservative management without surgical decompression despite large collection size.

## Case description

A 76-year-old male presented to our institution with dyspnoea. He had history of two-vessel coronary artery bypass grafting (CABG) 7 years prior, with left internal mammary artery (LIMA) to left anterior descending (LAD) and a saphenous vein graft (SVG) to ramus intermedius (RI) vessel. He also had previous PCI 1 year before with trifurcation drug-eluting stents placed at the junction of the left main (LM), RI, and proximal left circumflex coronary (LCx) arteries.

Two weeks prior, he had presented to an outside institution with chest pain in the setting of NSTEMI. He was taken for emergent angiogram and initially had two drug-eluting stents to ostial RCA stenosis and was also shown to have chronic severe in-stent restenosis of the trifurcation stents, occluded LAD with widely patent LIMA graft but a complete total occlusion (CTO) at LIMA touchdown, and occluded SVG to RI.

He then proceeded to have staged complex PCI 1 week later including laser atherectomy and balloon angioplasty of the LM, RI, and LCx, as well as multiple unsuccessful attempts at PCI of the occluded LAD via retrograde wiring from the LIMA. Immediate post-procedure angiography demonstrated no dissection or perforation, and transthoracic echocardiogram (TTE) 24 h after the second PCI showed no pericardial effusion, and the patient was discharged.

**Figure 1:**
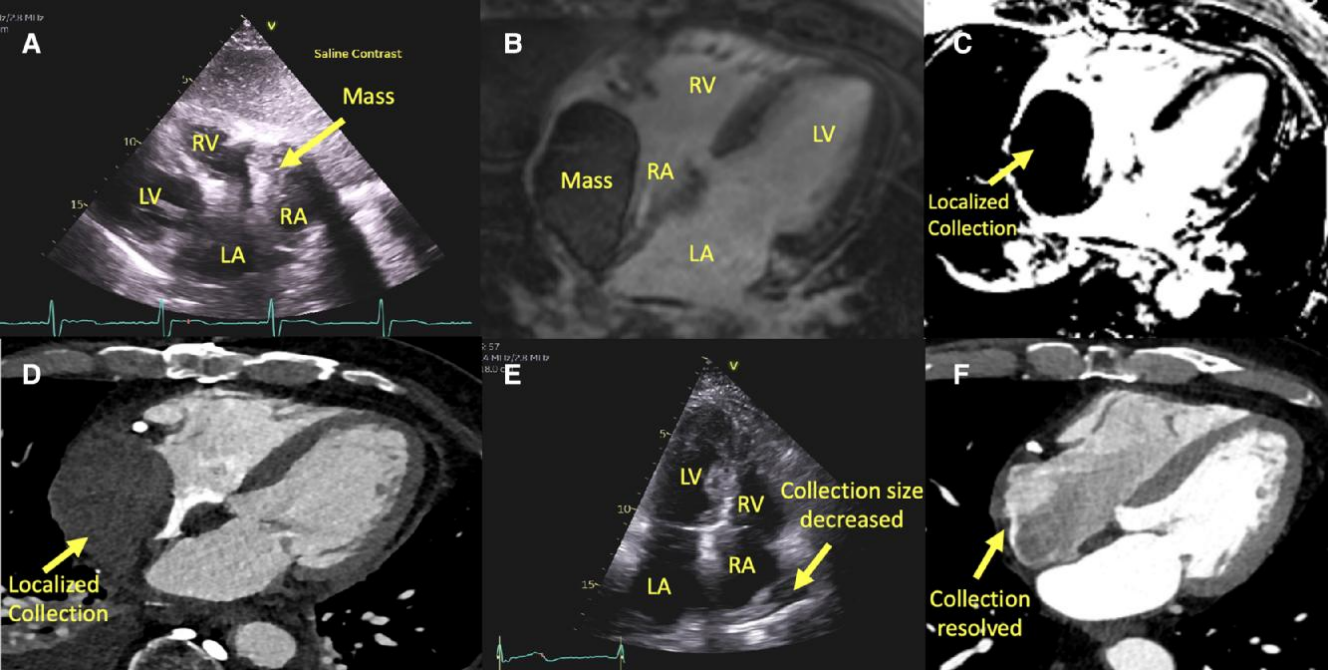
Multimodality imaging. RV, right ventricle; LV, left ventricle; LA, left atrium; RA, right atrium.

On presentation 1 week after the staged complex PCI, he was haemodynamically stable with heart rate 65 b.p.m. and blood pressure 140/64 mmHg. Physical exam revealed no murmurs or signs of heart failure. Transthoracic echocardiogram revealed a dense mass-like structure either in the right atrium (RA) or extrinsically compressing it (*Figure 1A*; Supplementary material online, *Videos S1* and *S2*). There was no pericardial effusion evident elsewhere, and no right-sided chamber collapse in diastole. Cardiac MRI was performed to help distinguish between differentials of thrombus, tumour, pseudoaneurysm, or pericardial haematoma and showed large extrinsic intrapericardial mass (9 × 5 × 8 cm; *Figure 1B*) with heterogeneously increased signal on T_1_- and T_2_-weighted sequences and did not perfuse with gadolinium (*Figure 1C*). Tissue characterization features and temporal course were consistent with a diagnosis of subacute haematoma due to CAP post PCI. Whereas acute haematomas have homogenous and high intensity signals, subacutely more heterogeneity and a dark peripheral rim may be observed.^1^ Lack of gadolinium enhancement excluded vascularity seen in a neoplastic process and suggested no active extravasation. Imaging features were not consistent with thrombus or pseudoaneurysm.

Despite low incidence of CAP, highest risk occurs with complex PCI, leading to pericardial collection.^2^ There is no current guideline consensus on CAP treatment; however, it is vital to seal the extravasation site and intervene quickly in the case of haemodynamic instability from ensuing pericardial effusion and tamponade. Various modalities have been proposed including prolonged balloon inflation at the designated site while ensuring distal coronary perfusion, reversal of anticoagulation in the setting of a large CAP, covered stents for proximal CAP, coil embolization for more distal CAP, and pericardiocentesis or emergent surgery for persistent haemodynamic embarrassment.^3^

The haematoma was likely caused by instrumentation in the second staged coronary procedure to the left coronary system that attempted to open the occluded native LAD with wiring of the LIMA and was focally contained likely due to adhesions from prior CABG. Surgical decompression was considered; however, conservative management with surveillance was undertaken given haemodynamic stability without tamponade physiology. Cardiac CT was obtained a few days after to re-evaluate and ensure no increase in collection size (*Figure 1D*). Transthoracic echocardiogram 6 weeks later showed significant reduction in size (*Figure 1E*; Supplementary material online, *Videos S3* and *S4*), and repeat CT 3 months later demonstrated complete resolution of the pericardial haematoma (*Figure 1F*). This case highlights the potential for a late presentation of CAP and successful conservative management despite large collection given absence of tamponade.

## Supplementary Material

ytae470_Supplementary_Data

## Data Availability

The data supporting the findings displayed in the case study are readily available from the corresponding author upon reasonable request.

## Supplementary material

**Consent:** The authors confirm that informed consent for submission and publication, including the use of imaging and video clips, was obtained from the patient for publication as per the COPE guidelines. All patient identifiers have been removed from the images.

**Funding:** None declared.
